# Supplementary material for: Weak spatiotemporal response of prey to predation risk in a freely interacting system
Source: J Anim Ecol. 2019 Mar 21;89(1):120–31. doi: 10.1111/1365-2656.12968 (PMC7003944; doi:10.1111/1365-2656.12968)
Supplement: Supplementary file 1 [file JANE-89-120-s001.docx]

**Figure S1.** Distribution of elk GPS fix intervals for the four winters considered in this study.
