# Supplementary material for: Weak spatiotemporal response of prey to predation risk in a freely interacting system
Source: J Anim Ecol. 2019 Mar 21;89(1):120–31. doi: 10.1111/1365-2656.12968 (PMC7003944; doi:10.1111/1365-2656.12968)
Supplement: Supplementary file 4 [file JANE-89-120-s004.pdf]

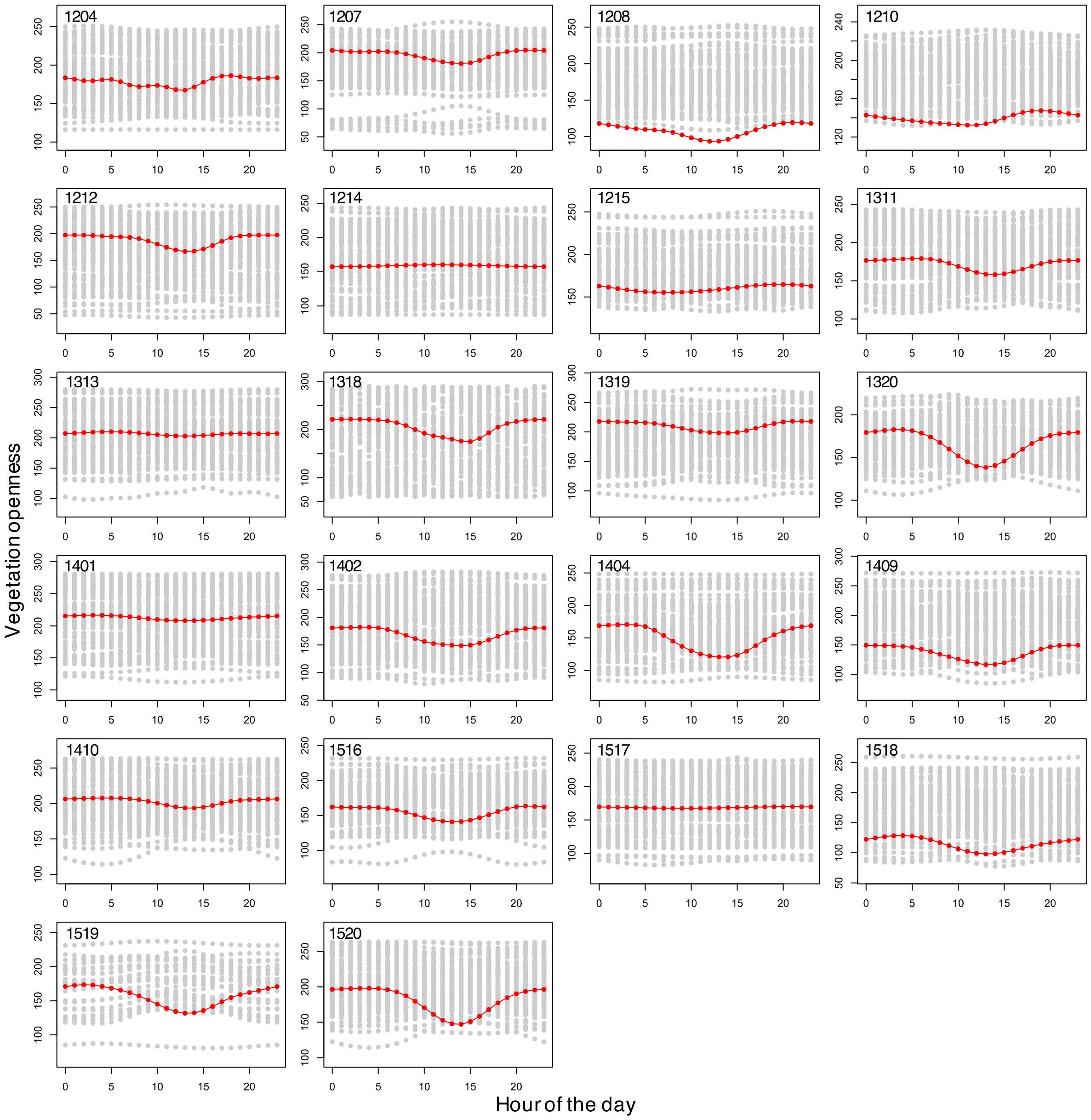

Figure S4. Observed (red line and full circles) and expected values (grey full circles) of vegetation openness for each hour of the day, as estimated from generalised additive mixed models. Each panel represents results for a single elk during the winter of 2014-15.
