# Supplementary material for: Weak spatiotemporal response of prey to predation risk in a freely interacting system
Source: J Anim Ecol. 2019 Mar 21;89(1):120–31. doi: 10.1111/1365-2656.12968 (PMC7003944; doi:10.1111/1365-2656.12968)
Supplement: Supplementary file 10 [file JANE-89-120-s010.docx]

Table S5. Details of individual elk trajectories and observed and expected encounter rates defined using a distance threshold of 500 m during the six winter periods considered (2013-2015). Expected values were obtained from a null model formulation that randomised elk trajectories within the same bounding box as the original trajectory, thus accounting for philopatric behaviour.

| Winter period | Elk ID | # relocations | Mean # hours  between relocations | # encounters | Encounter rate per 100 fixes | # days per encounter | Null model | | |
| --- | --- | --- | --- | --- | --- | --- | --- | --- | --- |
|  |  |  |  |  |  |  | Exp. | Var. | *P* |
| LW 2013 | 1201 | 307 | 2.497 | 0 | 0.000 | - | 0.130 | 0.094 | 0.572 |
|  | 1202 | 307 | 2.497 | 0 | 0.000 | - | 0.000 | 0.000 | 1.000 |
|  | 1203 | 306 | 2.505 | 1 | 0.327 | 30.0 | 0.324 | 0.238 | 0.543 |
|  | 1204 | 306 | 2.505 | 0 | 0.000 | - | 0.337 | 0.549 | 0.483 |
|  | 1205 | 270 | 2.827 | 0 | 0.000 | - | 0.278 | 0.267 | 0.483 |
|  | 1206 | 306 | 2.505 | 0 | 0.000 | - | 0.248 | 0.279 | 0.523 |
|  | 1207 | 307 | 2.497 | 0 | 0.000 | - | 0.619 | 1.926 | 0.374 |
|  | 1208 | 307 | 2.497 | 0 | 0.000 | - | 0.355 | 0.710 | 0.533 |
|  | 1209 | 307 | 2.497 | 2 | 0.651 | 15.0 | 0.205 | 0.128 | 0.731 |
|  | 1210 | 288 | 2.662 | 0 | 0.000 | - | 0.378 | 0.487 | 0.414 |
|  | 1211 | 306 | 2.507 | 0 | 0.000 | - | 0.444 | 0.260 | 0.236 |
|  | 1212 | 302 | 2.538 | 0 | 0.000 | - | 0.003 | 0.001 | 0.790 |
|  | 1214 | 306 | 2.505 | 2 | 0.654 | 15.0 | 0.170 | 0.312 | 0.750 |
|  | 1215 | 307 | 2.497 | 0 | 0.000 | - | 0.658 | 1.217 | 0.246 |
|  | 1311 | 306 | 2.505 | 1 | 0.327 | 30.0 | 0.252 | 0.330 | 0.642 |
|  | 1312 | 307 | 2.505 | 0 | 0.000 | - | 0.150 | 0.151 | 0.612 |
|  | 1313 | 307 | 2.505 | 0 | 0.000 | - | 0.283 | 0.194 | 0.394 |
|  | 1314 | 307 | 2.505 | 3 | 0.977 | 10.0 | 0.710 | 1.304 | 0.582 |
|  | 1315 | 307 | 2.505 | 0 | 0.000 | - | 0.309 | 0.387 | 0.483 |
|  | 1316 | 307 | 2.505 | 0 | 0.000 | - | 0.899 | 2.144 | 0.364 |
|  | 1317 | 307 | 2.505 | 0 | 0.000 | - | 0.007 | 0.002 | 0.780 |
|  | 1318 | 307 | 2.505 | 1 | 0.326 | 30.0 | 1.179 | 2.302 | 0.275 |
|  | 1319 | 307 | 2.498 | 0 | 0.000 | - | 0.124 | 0.195 | 0.651 |
|  | 1320 | 306 | 2.513 | 4 | 1.307 | 7.5 | 0.320 | 0.326 | 0.741 |
| EW 2013 | 1202 | 308 | 2.500 | 0 | 0.000 | - | 0.016 | 0.011 | 0.770 |
|  | 1204 | 308 | 2.500 | 3 | 0.974 | 10.0 | 0.724 | 0.649 | 0.572 |
|  | 1205 | 308 | 2.500 | 0 | 0.000 | - | 0.536 | 0.595 | 0.196 |
|  | 1207 | 308 | 2.500 | 0 | 0.000 | - | 0.610 | 1.749 | 0.275 |
|  | 1210 | 297 | 2.593 | 2 | 0.673 | 15.0 | 0.582 | 0.332 | 0.543 |
|  | 1212 | 308 | 2.500 | 7 | 2.273 | 4.3 | 0.503 | 0.663 | 0.770 |
|  | 1214 | 285 | 2.703 | 0 | 0.000 | - | 0.326 | 0.207 | 0.305 |
|  | 1215 | 308 | 2.500 | 1 | 0.325 | 30.0 | 0.555 | 0.375 | 0.384 |
|  | 1311 | 364 | 2.114 | 1 | 0.275 | 30.0 | 0.255 | 0.182 | 0.552 |
|  | 1312 | 308 | 2.500 | 0 | 0.000 | - | 0.494 | 0.408 | 0.246 |
|  | 1313 | 298 | 2.584 | 0 | 0.000 | - | 0.121 | 0.056 | 0.552 |
|  | 1314 | 308 | 2.500 | 0 | 0.000 | - | 0.481 | 0.423 | 0.246 |
|  | 1315 | 302 | 2.550 | 0 | 0.000 | - | 0.470 | 0.532 | 0.285 |
|  | 1316 | 308 | 2.500 | 0 | 0.000 | - | 0.373 | 0.229 | 0.246 |
|  | 1317 | 308 | 2.500 | 1 | 0.325 | 30.0 | 0.292 | 0.246 | 0.612 |
|  | 1318 | 308 | 2.500 | 0 | 0.000 | - | 0.094 | 0.086 | 0.691 |
|  | 1319 | 308 | 2.500 | 0 | 0.000 | - | 0.334 | 0.346 | 0.394 |
|  | 1320 | 371 | 2.074 | 1 | 0.270 | 30.0 | 0.434 | 0.348 | 0.414 |
| LW 2014 | 1202 | 306 | 2.505 | 0 | 0.000 | - | 0.000 | 0.000 | 1.000 |
|  | 1204 | 306 | 2.505 | 0 | 0.000 | - | 0.268 | 0.318 | 0.523 |
|  | 1205 | 306 | 2.505 | 0 | 0.000 | - | 0.163 | 0.165 | 0.572 |
|  | 1207 | 306 | 2.505 | 0 | 0.000 | - | 0.029 | 0.020 | 0.750 |
|  | 1208 | 306 | 2.505 | 5 | 1.634 | 6.0 | 1.020 | 1.951 | 0.602 |
|  | 1210 | 287 | 2.671 | 0 | 0.000 | - | 0.589 | 0.693 | 0.216 |
|  | 1212 | 306 | 2.505 | 0 | 0.000 | - | 0.078 | 0.147 | 0.721 |
|  | 1214 | 306 | 2.505 | 2 | 0.654 | 15.0 | 0.621 | 0.713 | 0.503 |
|  | 1215 | 305 | 2.513 | 0 | 0.000 | - | 0.246 | 0.255 | 0.523 |
|  | 1311 | 306 | 2.505 | 3 | 0.980 | 10.0 | 0.873 | 2.542 | 0.572 |
|  | 1312 | 305 | 2.513 | 0 | 0.000 | - | 0.443 | 0.589 | 0.364 |
|  | 1313 | 306 | 2.505 | 0 | 0.000 | - | 0.183 | 0.128 | 0.473 |
|  | 1314 | 306 | 2.505 | 0 | 0.000 | - | 0.408 | 0.311 | 0.325 |
|  | 1315 | 299 | 2.564 | 1 | 0.334 | 30.0 | 0.662 | 1.125 | 0.434 |
|  | 1317 | 306 | 2.505 | 0 | 0.000 | - | 0.866 | 1.058 | 0.147 |
|  | 1318 | 306 | 2.505 | 1 | 0.327 | 30.0 | 0.157 | 0.204 | 0.691 |
|  | 1319 | 306 | 2.505 | 3 | 0.980 | 10.0 | 0.062 | 0.021 | 0.800 |
|  | 1320 | 306 | 2.505 | 0 | 0.000 | - | 0.859 | 1.583 | 0.176 |
|  | 1401 | 307 | 2.497 | 0 | 0.000 | - | 0.007 | 0.002 | 0.780 |
|  | 1402 | 307 | 2.497 | 5 | 1.629 | 6.0 | 0.446 | 0.407 | 0.731 |
|  | 1404 | 307 | 2.497 | 0 | 0.000 | - | 0.189 | 0.170 | 0.543 |
|  | 1409 | 300 | 2.555 | 2 | 0.667 | 15.0 | 0.473 | 0.645 | 0.622 |
|  | 1410 | 307 | 2.497 | 0 | 0.000 | - | 0.088 | 0.079 | 0.681 |
| EW 2014 | 1202 | 304 | 2.533 | 1 | 0.329 | 30.0 | 0.128 | 0.096 | 0.691 |
|  | 1204 | 308 | 2.500 | 1 | 0.325 | 30.0 | 0.367 | 0.274 | 0.523 |
|  | 1207 | 308 | 2.500 | 0 | 0.000 | - | 0.403 | 0.411 | 0.265 |
|  | 1210 | 291 | 2.647 | 0 | 0.000 | - | 0.330 | 0.272 | 0.325 |
|  | 1212 | 289 | 2.665 | 0 | 0.000 | - | 0.318 | 0.476 | 0.493 |
|  | 1214 | 284 | 2.703 | 2 | 0.704 | 15.0 | 0.588 | 0.481 | 0.533 |
|  | 1215 | 280 | 2.751 | 0 | 0.000 | - | 0.429 | 0.438 | 0.335 |
|  | 1313 | 308 | 2.500 | 0 | 0.000 | - | 0.578 | 0.842 | 0.255 |
|  | 1314 | 308 | 2.500 | 0 | 0.000 | - | 0.175 | 0.114 | 0.473 |
|  | 1315 | 308 | 2.500 | 3 | 0.974 | 10.0 | 0.510 | 0.475 | 0.681 |
|  | 1318 | 308 | 2.500 | 5 | 1.623 | 6.0 | 0.734 | 0.891 | 0.651 |
|  | 1319 | 308 | 2.500 | 1 | 0.325 | 30.0 | 0.354 | 0.428 | 0.562 |
|  | 1320 | 308 | 2.500 | 4 | 1.299 | 7.5 | 0.344 | 0.264 | 0.770 |
|  | 1401 | 308 | 2.500 | 0 | 0.000 | - | 0.458 | 0.407 | 0.206 |
|  | 1402 | 308 | 2.500 | 3 | 0.974 | 10.0 | 0.484 | 0.548 | 0.681 |
|  | 1404 | 308 | 2.500 | 0 | 0.000 | - | 0.354 | 0.349 | 0.354 |
|  | 1409 | 308 | 2.500 | 0 | 0.000 | - | 0.487 | 0.491 | 0.265 |
|  | 1410 | 308 | 2.500 | 0 | 0.000 | - | 0.399 | 0.304 | 0.325 |
| LW 2015 | 1202 | 300 | 2.555 | 0 | 0.000 | - | 0.000 | 0.000 | 1.000 |
|  | 1204 | 307 | 2.497 | 1 | 0.326 | 30.0 | 0.528 | 0.413 | 0.453 |
|  | 1208 | 307 | 2.497 | 0 | 0.000 | - | 0.202 | 0.139 | 0.483 |
|  | 1210 | 289 | 2.653 | 0 | 0.000 | - | 0.211 | 0.193 | 0.572 |
|  | 1212 | 301 | 2.547 | 0 | 0.000 | - | 0.000 | 0.000 | 1.000 |
|  | 1214 | 307 | 2.497 | 0 | 0.000 | - | 0.567 | 0.822 | 0.246 |
|  | 1215 | 295 | 2.599 | 0 | 0.000 | - | 0.298 | 0.256 | 0.354 |
|  | 1311 | 295 | 2.599 | 2 | 0.678 | 15.0 | 0.776 | 0.839 | 0.473 |
|  | 1314 | 307 | 2.497 | 0 | 0.000 | - | 0.000 | 0.000 | 1.000 |
|  | 1318 | 307 | 2.497 | 4 | 1.303 | 7.5 | 1.039 | 0.962 | 0.463 |
|  | 1319 | 307 | 2.497 | 0 | 0.000 | - | 0.104 | 0.070 | 0.622 |
|  | 1320 | 306 | 2.505 | 3 | 0.980 | 10.0 | 0.980 | 1.238 | 0.483 |
|  | 1401 | 307 | 2.497 | 1 | 0.326 | 30.0 | 0.173 | 0.277 | 0.711 |
|  | 1402 | 307 | 2.497 | 0 | 0.000 | - | 0.267 | 0.387 | 0.562 |
|  | 1404 | 307 | 2.497 | 0 | 0.000 | - | 0.111 | 0.118 | 0.651 |
|  | 1409 | 307 | 2.497 | 0 | 0.000 | - | 0.127 | 0.146 | 0.651 |
|  | 1410 | 294 | 2.607 | 0 | 0.000 | - | 0.194 | 0.199 | 0.552 |
|  | 1516 | 307 | 2.497 | 2 | 0.651 | 15.0 | 0.863 | 1.017 | 0.453 |
|  | 1517 | 307 | 2.497 | 4 | 1.303 | 7.5 | 0.638 | 0.602 | 0.651 |
|  | 1518 | 296 | 2.590 | 0 | 0.000 | - | 0.307 | 0.418 | 0.453 |
|  | 1519 | 307 | 2.497 | 0 | 0.000 | - | 0.261 | 0.234 | 0.453 |
|  | 1520 | 307 | 2.497 | 0 | 0.000 | - | 0.114 | 0.097 | 0.622 |
| EW 2015 | 1208 | 298 | 2.584 | 0 | 0.000 | - | 0.044 | 0.013 | 0.671 |
|  | 1210 | 286 | 2.693 | 1 | 0.350 | 30.0 | 0.483 | 0.597 | 0.533 |
|  | 1311 | 304 | 2.533 | 0 | 0.000 | - | 0.076 | 0.032 | 0.622 |
|  | 1314 | 324 | 2.375 | 1 | 0.309 | 30.0 | 0.074 | 0.070 | 0.741 |
|  | 1317 | 314 | 2.450 | 1 | 0.318 | 30.0 | 0.131 | 0.074 | 0.701 |
|  | 1318 | 331 | 2.324 | 0 | 0.000 | - | 0.000 | 0.000 | 1.000 |
|  | 1319 | 308 | 2.500 | 1 | 0.325 | 30.0 | 0.328 | 0.295 | 0.552 |
|  | 1320 | 312 | 2.466 | 0 | 0.000 | - | 0.314 | 0.353 | 0.453 |
|  | 1401 | 324 | 2.375 | 0 | 0.000 | - | 0.028 | 0.010 | 0.721 |
|  | 1404 | 311 | 2.474 | 0 | 0.000 | - | 0.244 | 0.234 | 0.434 |
|  | 1410 | 317 | 2.427 | 0 | 0.000 | - | 0.123 | 0.132 | 0.651 |
|  | 1516 | 314 | 2.450 | 0 | 0.000 | - | 0.194 | 0.446 | 0.602 |
|  | 1517 | 332 | 2.317 | 0 | 0.000 | - | 0.117 | 0.093 | 0.622 |
|  | 1518 | 305 | 2.520 | 1 | 0.328 | 30.0 | 0.144 | 0.185 | 0.701 |
|  | 1519 | 311 | 2.474 | 0 | 0.000 | - | 0.257 | 0.163 | 0.364 |
